# Predictors of the Fear of COVID-19 in Individuals with Schizophrenia: a Prospective Study

**DOI:** 10.7150/ijms.106953

**Published:** 2025-03-21

**Authors:** Cheng-Fang Yen, Ching-Shu Tsai, Chien-Wen Lin, Ray C. Hsiao, Peng-Wei Wang

**Affiliations:** 1 Department of Psychiatry, Kaohsiung Medical University Hospital, Kaohsiung, Taiwan.; 2 Department of Psychiatry, School of Medicine, College of Medicine, Kaohsiung Medical University, Kaohsiung, Taiwan.; 3 College of Professional Studies, National Pingtung University of Science and Technology, Pingtung, Taiwan.; 4 Department of Child and Adolescent Psychiatry, Chang Gung Memorial Hospital, Kaohsiung Medical Center, Kaohsiung, Taiwan.; 5 School of Medicine, Chang Gung University, Taoyuan, Taiwan.; 6 Department of Psychiatry, Kaohsiung Medical University Gangshan Hospital, Kaohsiung, Taiwan.; 7 Department of Psychiatry, Seattle Children's Hospital, Seattle, WA, USA.; 8 Department of Psychiatry and Behavioral Sciences, School of Medicine, University of Washington, Seattle, WA, USA.

**Keywords:** COVID-19, schizophrenia, stigma, fear of COVID-19, psychological well-being

## Abstract

**Purpose:** The fear of COVID-19 can result in psychological distress and mental health problems. Individuals with schizophrenia (IWSs) are especially vulnerable to contracting COVID-19. This study with a 1-year follow-up examined whether individual characteristics (sociodemographic characteristics, schizophrenia symptoms, depression, and self-esteem) or factors related to individual-environmental interaction (self-stigma, loneliness, and perceived social support) predicted the level of fear of COVID-19 (FC) among IWSs.

**Patients and methods:** In total, 257 IWSs (out of an initial pool of 300 IWSs) were enrolled, and their baseline data were collected. FC was assessed using the Fear of COVID-19 Scale at 1 year after the onset of the study. The associations of baseline factors with FC 1 year later were analyzed using bivariable and multiple linear regression analysis.

**Results:** Bivariable linear regression results demonstrated that being a woman (*p* < 0.05), being unemployed (*p* < 0.05), having depression (*p* < 0.001), having low self-esteem (*p* < 0.05), experiencing loneliness (*p* < 0.01), and having feelings of self-stigma (*p* < 0.001) significantly predicted FC 1 year later. A multiple linear regression model further indicated that having feelings of self-stigma significantly predicted FC 1 year later (*p* < 0.01).

**Conclusion:** Clinicians and policymakers should consider the predictors identified in this study when designing interventions to reduce FC among IWSs.

## Introduction

Fear is the response to the existence of a threat and generally drives actions toward self-protection 1. Fear can lead individuals to behave in a dysfunctional manner, resulting in the development of general distress and mental health problems 2, 3. The fear of respiratory infectious diseases (RIDs), including COVID-19 is an unpleasant emotional state that is elicited by the perception of danger and insecurity feelings in the RIDs epidemics or pandemics 4. People develop the fear of RIDs because of not only the high transmission rate and severe consequences of contraction but also the control measures such as quarantine for prevention of pathogens spreading 5. A meta-analysis found that fear of COVID-19 (FC) has moderate associations with mental health problems such as anxiety, depression, and sleep problems 2. FC also makes an individual less able and motivated to adopt preventive behaviors against COVID-19 6. Therefore, health-care professionals must not neglect FC when providing medical services in the aftermath of the COVID-19 pandemic.

Individuals with schizophrenia (IWSs) have higher risks of contracting COVID-19 and hospitalization and mortality due to contracting COVID-19 than individuals in the general population 7-11. Previous studies have found that FC increases anxiety, loneliness, and depression in individuals with schizophrenia 12, 13. Multiple COVID-19 related factors contribute to the development of FC, such as perceived vulnerability to and harms of contracting COVID-19, pandemic prevention policies (such as quarantine), a lack of pandemic-related information, and contradictions in the public statements of experts and authorities 14. However, according to ecological system theory 15, FC result from the interactions between individuals and their environment. Several studies have identified individual factors contributing to FC in IWSs, including gender (women reported greater FC than men) 16 and income (higher income was negatively associated with FC) 17. Additionally, having psychotic symptoms was significantly associated with FC in IWSs 18.

Whether variables related to the interaction of individual and environmental factors (referred to as individual-environmental factors for brevity) such as self-stigma, loneliness, and perceived social support can predict the level of FC in IWSs has not been thoroughly examined. Self-stigma refers to how individuals perceive, accept, and internalize negative stereotypes from others in their attitude toward themselves 19, 20. Self-stigma can negatively affect the mood of IWSs and increase the severity of their depression, anxiety, and sense of hopelessness; these, in turn, reduce their willingness to follow medical advice 21-23. The use of self-stigma as a predictive factor in the development of FC among IWSs has not been thoroughly explored. Loneliness refers to the perceived gap between anticipated and actual levels of social connectivity 24. Previous cross-sectional studies in IWSs have observed that loneliness was positively associated with FC 13, whereas perceived social support was negatively associated with distress during the COVID-19 pandemic 12. Whether loneliness and perceived social support can predict the degree of the FC in IWSs requires examination in longitudinal studies. Further, low self-esteem is associated with perceived threats and fear arousal in the general population 25. Self-esteem refers to acceptance and respect for oneself based on the instinctive need to be valued as human being 26. One study found that self-esteem protected against anxiety symptoms triggered by FC 27. However, self-esteem has not been extensively explored as a predictor of FC in IWSs.

The present study followed the IWSs participating in a previous study conducted to determine the experiences of stigma and mental health before the severe outbreak of COVID-19 in Taiwan 28-30 and examined the performance of baseline individual characteristics (sociodemographic characteristics, symptoms of schizophrenia, and self-esteem) and individual-environmental factors (self-stigma, loneliness, and perceived social support) as predictors of FC among IWSs one year later. We hypothesized that, among IWSs, FC is (i) associated with a variety of sociodemographic characteristics and schizophrenia symptoms; (ii) positively associated with greater depressive symptoms, self-stigma, and loneliness; and (iii) negatively associated with greater self-esteem and perceived social support.

## Material and methods

### Study Participants

The present study followed 300 IWSs participating in a study examining the experiences of stigma and mental health 28-31 and examined the predictive effects of baseline individual and individual-environmental factors on FC one year later. The original study initially collected data on 300 IWSs recruited from two community psychiatric rehabilitation institutes and the psychiatric outpatient clinic of a university hospital over the period from February 2022 to May 2022 28-31. Taiwan had a relatively mild outbreak of COVID-19 before February 2022; only 0.077% people in Taiwan had been infected with COVID-19 prior this study 32. Therefore, the baseline data presented the situation of the vast majority of Taiwanese IWSs before the outbreak of COVID-19. The diagnosis of schizophrenia was made according to the criteria of the Diagnostic and Statistical Manual of Mental Disorders, Fifth Edition 33. One year later, the same 300 individuals were invited to participate in this follow-up study. Trained research assistants explained the purpose and procedure of the follow-up study to the participants. After providing written informed consent, the participants underwent a face-to-face interview in interview rooms for their level of FC to be assessed.

### Ethics statement

This study was approved by the institutional review board of Kaohsiung Medical University Hospital (KMUHIRB-SV(I)-20220063). The participants provided their written informed consent to participate in this study. This questionnaire study did not conduct experiments on humans or human tissue samples. This study conformed to the Declaration of Helsinki and the Recommendations for the Conduct, Reporting, Editing, and Publication of Scholarly Work in Medical Journals.

### Measures

#### Predictors at baseline

Participants' sociodemographic characteristics, psychiatric diagnosis and symptoms, self-esteem, self-stigma, loneliness, and perceived social support were assessed at baseline.

#### Sociodemographic characteristics

The participants' gender (female vs*.* male), age, total years of education completed, total monthly income, marital status (separated or divorced vs*.* married or cohabiting), and occupational status (unemployed vs. employed) were collected.

#### Psychiatric symptoms of schizophrenia

Participants' duration of illness (defined as the total years since the initial diagnosis of schizophrenia) was collected. Psychiatrists assessed participants along five domains of psychiatric symptoms, comprising positive symptoms, negative symptoms, disorganization, excitement, and emotional distress, using the Chinese version of the Positive and Negative Syndrome Scale (C-PANSS) 34-36. The PANSS is the commonly used tool for assessing psychotic symptoms of patients with schizophrenia 35, 36. Each item on the C-PANSS for the severity of psychiatric symptoms is rated on a 7-point scale from 1 (*absent*) to 7 (*extreme*). A higher mean score for each domain indicates more severe psychiatric symptoms. The raters received standardized training for rating the severity of psychiatric symptoms on the C-PANSS prior to the study; the inter-rater reliability (Cohen's kappa coefficient) of the C-PANSS was 0.85. Cronbach's α for the five modules in our study ranged from 0.65 to 0.80. Emotional distress substantially overlapped with depressive symptoms and thus was not used in any linear regression model to reduce the level of collinearity. Participants reported their frequency of depressive symptoms in the preceding month on the Chinese version of the Center for Epidemiologic Studies—Depression Scale (MC-CES-D) 37, 38. Each item on the MC-CES-D is rated on a 4-point scale from 0 (*rarely or none of the time*) to 3 (*most or all of the time*). A higher total MC-CES-D score indicates more severe depressive symptoms. The MC-CES-D has satisfactory internal consistency (Cronbach's α = 0.90), 1-week test-retest reliability (intraclass correlation reliability = 0.93), congruent validity (area under the receiver operative characteristic curves for major depressive disorder = 0.88-0.90), and construct validity 39, 40. Cronbach's α in our study was 0.91.

#### Self-esteem

Participants rated their global self-esteem using the Mandarin Chinese version of the Rosenberg Self-Esteem Scale (M-RSES) 41. The RSES is one of the commonly used instruments for assessing the level of self-esteem. Each item on the M-RSES is rated on a 4-point scale from 1 (*strongly disagree*) to 4 (*strongly agree*). A higher M-RSES total score indicates a higher level of global self-esteem. This scale has high reliability and construct validity 41. Cronbach's alpha in this study was 0.84.

#### Self-stigma

Participants rated their level of self-stigma from having the diagnosis of schizophrenia on the Taiwanese version of the Self-Stigma Scale-Short (T-SSS-S) 42. The T-SSS-S is the first instrument for assessing the level of self-stigma in individuals with mental illnesses 50. Each item on the T-SSS-S is rated on a 4-point scale ranging from 1 (*strongly disagree*) to 4 (*strongly agree*). A higher total T-SSS-S score indicates a higher level of self-stigma toward having schizophrenia. The original version and Taiwanese version of the Self-Stigma Scale-Short were found to have acceptable internal consistency (Cronbach's alpha = 0.81-0.84 and 0.87, respectively) and satisfactory construct validity (comparative fit index = 0.97 and 0.99, respectively) in a confirmatory factor analysis model 42, 43. Cronbach's alpha in this study was 0.88.

#### Loneliness

Participants rated their degree of loneliness on the Chinese version of the University of California Los Angeles Loneliness Scale—Version 3 (C-UCLA-LS3) 44, 45. Each item on the C-UCLA-LS3 is rated on a 4-point scale from 1 (*never*) to 4 (*always*). A higher total C-UCLA-LS3 score indicates a higher level of loneliness. In previous research, the Chinese versions of the UCLA Loneliness Scale (Version 3) exhibited favorable psychometric properties among individuals with schizophrenia or schizoaffective disorder 44. Cronbach's α in our study was 0.90.

#### Perceived social support from families and friends

Participants rated the level of perceived social support from family members and friends on the Chinese version of the Adaptability, Partnership, Growth, Affection, and Resolve (C-APGAR) Index 46, 47. Each item on the C-APGAR is rated on a 4-point scale from 1 (*never*) to 4 (*always*). Higher total scores of the family and friend C-APGAR items indicate higher levels of perceived support from families and friends, respectively. Cronbach's alpha for the family and friend C-APGAR in this study was 0.94 and 0.93, respectively.

#### Outcome variable: FC

Participants rated their level of FC on the Fear of COVID-19 Scale (FCV-19S) 48. The FCV-19S is the instrument that have been used to assess FC of people across various countries, genders, age, and backgrounds (e.g., the general population, healthcare worker, and college students) in the COVID-19 pandemic 49. A review study examining the studies from 21 countries with 16 language versions of FCV-19S confirmed that the FCV-19S is a valid instrument for assessing fear across different languages 50. Ratings are given on a five-point Likert scale ranging from 1 (*strongly disagree*) to 5 (*strongly agree*). An analysis of several studies has independently validated the psychometric properties of FCV-19S obtained in samples of a generally reasonable size in diverse populations 51. The Taiwanese version of the FCV-19S has a single-factor structure with satisfactory fit indices; the fear of COVID-19 measured by the FCV-19S was significantly associated with psychological distress measured by Depression Anxiety Stress Scale-21 among individuals in Taiwan 6. Cronbach's alpha coefficient (α) of the FCV-19S in this study was 0.90.

### Statistical analysis

We conducted statistical analyses using IBM SPSS Statistics version 24.0 (International Business Machines Corporation, Armonk, NY, USA). Continuous variables are presented in terms of their mean and standard deviation; their distributions were tested for skewness and kurtosis 52. Categorical variables are presented in terms of their frequency and percentage. The present study used bivariable linear regression to examine the associations of factors at baseline with the reported FC at follow-up. Baseline factors significantly associated with FC at follow-up in bivariable models were utilized in a multivariable regression model. The potential multicollinearity was examined using variance inflation factors (VIF); a VIF value greater than 5 is considered indicative of significant multicollinearity 53. A p value lower than 0.05 indicated statistical significance.

## Results

In total, 257 IWSs (85.7% of the initial 300), of whom 55.6% were women, participated in the follow-up study; of the remaining initial IWSs, 13 (4.3%) refused to participate, and 30 (10%) were lost to follow-up. No significant differences in gender (p = 0.093), age (p = 0.389), or years of education (p = 0.459) were observed between those who participated in the follow-up study and those who did not. Table 1 presents the variables at baseline and FC at follow-up for the 257 participants.

Table 2 presents the results on the associations of factors at baseline with FC 1 year later. The unadjusted regression models demonstrated that female participants had greater FC than male participants (*p* < 0.05). Additionally, unemployed participants had greater FC than employed participants (*p* < 0.05). Moreover, higher levels of depression (*p* < 0.001), self-stigma (*p* < 0.001), and loneliness (*p* < 0.01) at baseline were significantly associated with greater FC 1 year later. By contrast, higher self-esteem at baseline was significantly associated with less FC one year later (*p* < 0.05).

Baseline factors significantly associated with FC were entered into a multivariable linear regression model, which indicated that greater self-stigma (*p* < 0.01) at baseline was significantly associated with greater FC 1 year later. All VIF values were less than 5, indicating no significant multicollinearity.

## Discussion

The adjusted regression model revealed that greater self-stigma at baseline was positively associated with FC in IWSs at follow-up 1 year later. The unadjusted regression models in this study also indicated that being female, being unemployed, having higher levels of depression, self-stigma, and loneliness, and having lower self-esteem at baseline were significantly associated with greater FC; however, the associations of these baseline variables with FC at follow-up became non-significant, indicating that there might be confounding effects among these variables.

The effect of self-stigma on FC in IWSs may stem from several mechanisms. First, self-stigma has substantial negative effects on the mental health of IWSs and increases the risks of depression, anxiety, hopelessness, and suicidality 21-23, 29. Mental health problems contribute to these individuals' automatic thoughts 54 and catastrophic beliefs 55, which are detrimental to rational appraisals of risk and timely responses to the urgent threat of COVID-19, potentially increasing FC in these individuals. Second, self-stigma and related mental health problems may compromise the self-efficacy of IWSs 56; this weakened self-efficacy may have increased the difficulty they experienced in confidently dealing with the problems encountered during the COVID-19 pandemic, exacerbating their FC. Third, self-stigma can worsen individuals' social functioning 57. A lack of connection with others may have limited information and psychological and material support available to IWSs during the COVID-19 pandemic, further exacerbating their FC. Fourth, self-stigma may decrease individuals' willingness to seek medical assistance from health-care professionals 57. Consequently, many IWSs experienced great FC during the pandemic due to a lack of reliable information and resources from medical professionals. The results of this study indicate that intervention programs for reducing self-stigma in IWSs are beneficial to the health of IWSs during normal times and can reduce fear during an outbreak of infectious diseases.

The unadjusted regression models in the present study demonstrated that loneliness but not perceived social support at baseline were positively associated with FC 1 year later in IWSs. Humans are innately social beings and have a neurobiological need to “belong to a certain group” 58, 59. Loneliness is the negative subjective feeling that arises when individuals are isolated, misunderstood, or rejected or when individuals are not satisfied with their social relationships 60. Loneliness is also an emotional consequence of the cognitive evaluation of social interactions. When expectations of the quality and quantity of social interactions and networks are unmet, individuals experience negative emotional reactions 61. According to social information processing models 62, 63, individuals with prominent loneliness are hypervigilant to socially threatening stimuli, expect to experience negative social interactions, reflexively make negative appraisals of self and others, remember more past negative messages than positive ones, and have future goals focused on preventing painful feelings rather than creating positive experiences 62-64. Therefore, during the pandemic, individuals who experienced considerable loneliness may have experienced great FC when faced with drastic changes to their routines and social interactions. Further, studies have found a positive association between loneliness and stigmatizing experiences in IWSs 65, 66. The association of loneliness and FC might be confounded by self-stigma in this study. Based on the results of this study, it is necessary to enhance social support for IWSs and help them adjust the perception of interpersonal interactions to reduce perceived loneliness.

The unadjusted regression models of the present study found that being a woman, being unemployed, feeling depressed, and having low self-esteem at baseline predicted FC 1 year later. Biological variables (e.g., hormones) and social factors (e.g., social expectations of gender roles) may account for the observed difference along gender lines in FC in IWSs 16. Employment provides a regular income, which reduced the negative influences of the COVID-19 pandemic on daily life and thus may have mitigated FC in employed IWSs 17. Depression can compromise cognitive function and skills that are essential to the adoption of protective behaviors against COVID-19, and may thus have increased FC in IWSs with depression. By contrast, IWSs with high self-esteem were likely to be more confident that they could manage the difficulties of COVID-19 and thus felt less threatened by the pandemic. Further, studies have found that both depressive symptoms and low self-esteem were associated with higher self-stigma in individuals with severe mental illnesses 23. Therefore, the associations of depressive symptoms and self-esteem with FC in IWSs might be confounded by self-stigma.

The present study demonstrated that self-stigma can predict FC among IWSs. Eliminating public stigma toward schizophrenia is essential to preventing the development of self-stigma. Therefore, government authorities and health-care professionals must educate the public on the etiology of schizophrenia and mitigate overt and subtle discrimination toward IWSs. For IWSs, psychoeducation and cognitive behavioral therapy effectively reduce self-stigma 67, 68. These intervention programs integrate elements of education about schizophrenia, cognitive techniques for dealing with public attitudes toward and beliefs in schizophrenia, and psychodynamic approach for dealing with emotional reactions associated with schizophrenia and stigma 67, 68. Several online intervention programs have been developed to improve the stigma in IWSs and should be feasible during a pandemic 69, 70. Community-based stigma reduction strategies, such as contact interventions, educational interventions, and family psychoeducation programs should be also established to reduce public stigma toward schizophrenia 71. Furthermore, although the predictive effects of individual factors (gender, employment status, depression, loneliness, and self-esteem) for FC did not reach significance in this study's multivariable regression model, health-care professionals must consider these variables when developing intervention programs to help IWSs cope with the changes caused by the COVID-19 pandemic.

This study has several limitations. First, the study collected self-reported data from IWSs and may be affected by informant bias. Second, the participants were invited from community psychiatric rehabilitation institutes and psychiatric outpatient clinics and were invested in participating in this follow-up assessment. Thus, the study may be affected by sampling bias. Third, this study did not examine the predictive effect of baseline physical health status and COVID-19-related variables such as participants' and family members' history of contracting COVID-19 and related hospitalization, receipt of COVID vaccination, COVID-related burden, and fear of COVID-19; these may be confounders affecting the study's results.

## Conclusion

Self-stigma significantly predicted the severity of FC 1 year later among IWSs. Gender, employment status, depression, loneliness, and self-esteem also had predictive effects on FC in bivariable linear regression models. The identified predictors should be considered in the design of interventions against FC among IWSs.

## Figures and Tables

**Table 1:** Participant characteristics (*n* = 257)

Variable	*n* (%)	Mean (SD)	Range
Gender			
Female	143 (55.6)		
Male	114 (44.4)		
Age (year)		45.6 (11.3)	20-70
Education (year)		13.0 (2.7)	6-20
Marital status			
Separated or divorced	222 (86.4)		
Married or cohabited	35 (13.6)		
Monthly disposable income (new Taiwan dollar)		7973.8 (8179.4)	0-60000
Occupation			
Unemployed	181 (70.4)		
Employed	76 (29.6)		
Duration of illness (year)		19.0 (10.0)	.50-43
Positive symptoms on the C-PANSS		3.5 (0.9)	2-6
Negative symptoms on the C-PANSS		3.6 (0.9)	1-6
Disorganization on the C-PANSS		3.5 (0.9)	1-6
Excitement on the C-PANSS		2.5 (0.9)	1-5
Depression		16.7 (11.1)	0-54
Self-esteem		28.1 (5.7)	11-40
Self-stigma		20.0 (5.3)	9-36
Loneliness		43.2 (11.5)	20-76
Perceived support from families		15.6 (3.7)	5-20
Perceived support from friends		13.5 (4.4)	5-20
Fear of COVID-19		18.9 (6.6)	7-35

COVID-19: Coronavirus Disease 2019; C-PANSS: Chinese version of Positive and Negative Syndrome Scale

**Table 2:** Associations of individual, environmental, and individual-environmental interaction factors with the fear of COVID-19 in linear regression analysis

	Unadjusted model	Adjusted model	
	*B (se)*	*B (se)*	VIF
Gender^a^	-1.740 (0.824)*	-1.554 (0.808)	1.030
Age	0.034 (0.036)	-	
Education	-0.193 (0.156)	-	
Monthly disposable income^b^	-0.028 (0.051)	-	
Marital status^c^	0.876 (1.203)	-	
Occupation^d^	-1.817 (0.898)*	-1.105 (0.887)	1.047
Duration of illness	0.028 (0.041)	-	
Positive symptoms on the C-PANSS	0.028 (0.463)	-	
Negative symptoms on the C-PANSS	0.575 (0.437)	-	
Disorganization on the C-PANSS	0.419 (0.474)	-	
Excitement on the C-PANSS	-0.001 (0.055)	-	
Depression	0.137 (0.036)***	0.079 (0.060)	2.829
Self-esteem	-0.165 (0.071)*	0.152 (0.106)	2.349
Self-stigma	0.331 (0.075)***	0.272 (0.104)**	1.947
Loneliness	0.093 (0.035)**	0.021 (0.050)	2.154
Perceived support from families	0.018 (0.113)	-	
Perceived support from friends	-0.138 (0.093)	-	

Reference criteria: ^a^ female, ^b^ 1000 new Taiwan dollars per unit, ^c^ separated or divorced, ^d^ unemployed. *p < .05; **p < .01; ***p < .001. COVID-19: Coronavirus Disease 2019; C-PANSS: Chinese version of Positive and Negative Syndrome Scale; VIF: Variance Inflation Factor

## Abbreviations

IWSs: Individuals with schizophrenia
COVID-19: Coronavirus Disease 2019
FC: fear of COVID-19
C-PANSS: Chinese version of the Positive and Negative Syndrome Scale
MC-CES-D: Chinese version of the Center for Epidemiologic Studies—Depression Scale
M-RSES: Mandarin Chinese version of the Rosenberg Self-Esteem Scale
T-SSS-S: Taiwanese version of the Self-Stigma Scale-Short
C-UCLA-LS3: Chinese version of the University of California Los Angeles Loneliness Scale—Version 3
C-APGAR: Chinese version of the Adaptability, Partnership, Growth, Affection, and Resolve Index
FCV-19S: Fear of COVID-19 Scale
